# Crystal structure and Hirshfeld surface analysis of bis­(3-aminopyrazole-κ*N* ^1^)bis­(3-aminopyrazole-κ*N* ^2^)bis­(nitrato-κ*O*)copper(II)

**DOI:** 10.1107/S2056989023009295

**Published:** 2023-10-31

**Authors:** Olesia I. Kucheriv, Irina A. Golenya, Olena Prysiazhna, Sofiia V. Partsevska, Il’ya A. Gural’skiy

**Affiliations:** a Department of Chemistry, Taras Shevchenko National University of Kyiv, Volodymyrska St. 64/13, Kyiv 01601, Ukraine; b Innovation Development Center ABN, Pirogov St. 2/37, 01030 Kyiv, Ukraine; c Bakul Institute for Superhard Materials, National Academy of Sciences of Ukraine, Avtozavodskaya St. 2, Kyiv 04074, Ukraine; d Department of Chemistry, Kyiv National University of Construction and Architecture, Povitroflotsky Ave. 31, Kyiv 03680, Ukraine; Vienna University of Technology, Austria

**Keywords:** crystal structure, pyrazole complex, copper(II), Jahn–Teller distortion

## Abstract

In the crystal structure of the title compound, [Cu(NO_3_)_2_(C_3_H_5_N_3_)_4_], the Cu^II^ atom is situated on an inversion center (Wyckoff position 2*c* of space group *P*2_1_/*n*) and shows an octa­hedral [N_4_O_2_] coordination environment. The axial positions are occupied by O atoms of nitrate anions, while the equatorial positions are taken up by the N atoms of four 3-amino­pyrazole ligands. As a result of the tautomerism of the latter, two coordinate with the N^1^-atom of 3-amino­pyrazole while the other two with the N^2^-atom. The presence of pyrrole-like N—H groups and amine substituents as donor groups leads to numerous intra- and inter­molecular hydrogen-bonding inter­actions, which were qu­anti­fied by Hirshfeld surface analysis.

## Abstract 2

The title mol­ecular coordination compound exhibits a central Cu^II^ atom with a distorted octa­hedral [N_4_O_2_] coordination environment. The axial positions are occupied by two O atoms from nitrate anions and equatorial positions occupied by pyridine-like N atoms from four 3-amino­pyrazole ligands.

## 1. Chemical context

Supra­molecular chemistry includes an extensive domain of pyrazole complexes, the main feature of which revolves around the formation of intra- and inter­molecular hydrogen bonds (Pérez & Riera, 2009 ▸). Pyrazole is a heterocyclic compound that contains two types of N atoms. One of the N atoms is termed pyridine-like because it donates one *p*-electron to the aromatic ring while its lone pair of electrons is non-conjugated. The other N atom is described as acidic pyrrole-like as it contributes two *p*-electrons of the lone pair to the aromatic ring, which consequently is distributed around the ring (Reedijk, 1987 ▸).

The presence of both pyridine-like and pyrrole-like N atoms makes a pyrazole mol­ecule both basic and acidic. With respect to the Brønsted–Lowry theory, this ligand is amphiprotic. Pyrazolate anions, which are the deprotonated form of pyrazole, form an individual class of ligands which, in contrast to pyrazole itself, can act as bridging. Apart from its ability to donate or accept a proton, an important feature of pyrazole is its tendency to from extensive networks of hydrogen bonds, in particular due to the simultaneous presence of a hydrogen-donating N—H group and a hydrogen-accepting pyridine-like N atom. The existence of these two groups allows the formation of inter­molecular N—H⋯N contacts and makes pyrazole an important mol­ecule for supra­molecular chemistry. In addition, numerous examples of practical applications have been offered for pyrazole-containing coordination compounds. For example, copper(II) complexes with pyrazole-containing ligands have been shown to exhibit catalytic (Gamez *et al.*, 2001 ▸; Titi *et al.*, 2023 ▸), anti­bacterial (Zaimović *et al.*, 2022 ▸), anti­fungal (Titi *et al.*, 2023 ▸) and anti­tumor (Ruan *et al.*, 2012 ▸) activities.

Here we describe the crystal structure of a new copper(II) complex with 3-amino­pyrazole as a ligand, namely [Cu(C_3_H_5_N_3_)_4_(NO_3_)_2_]. 
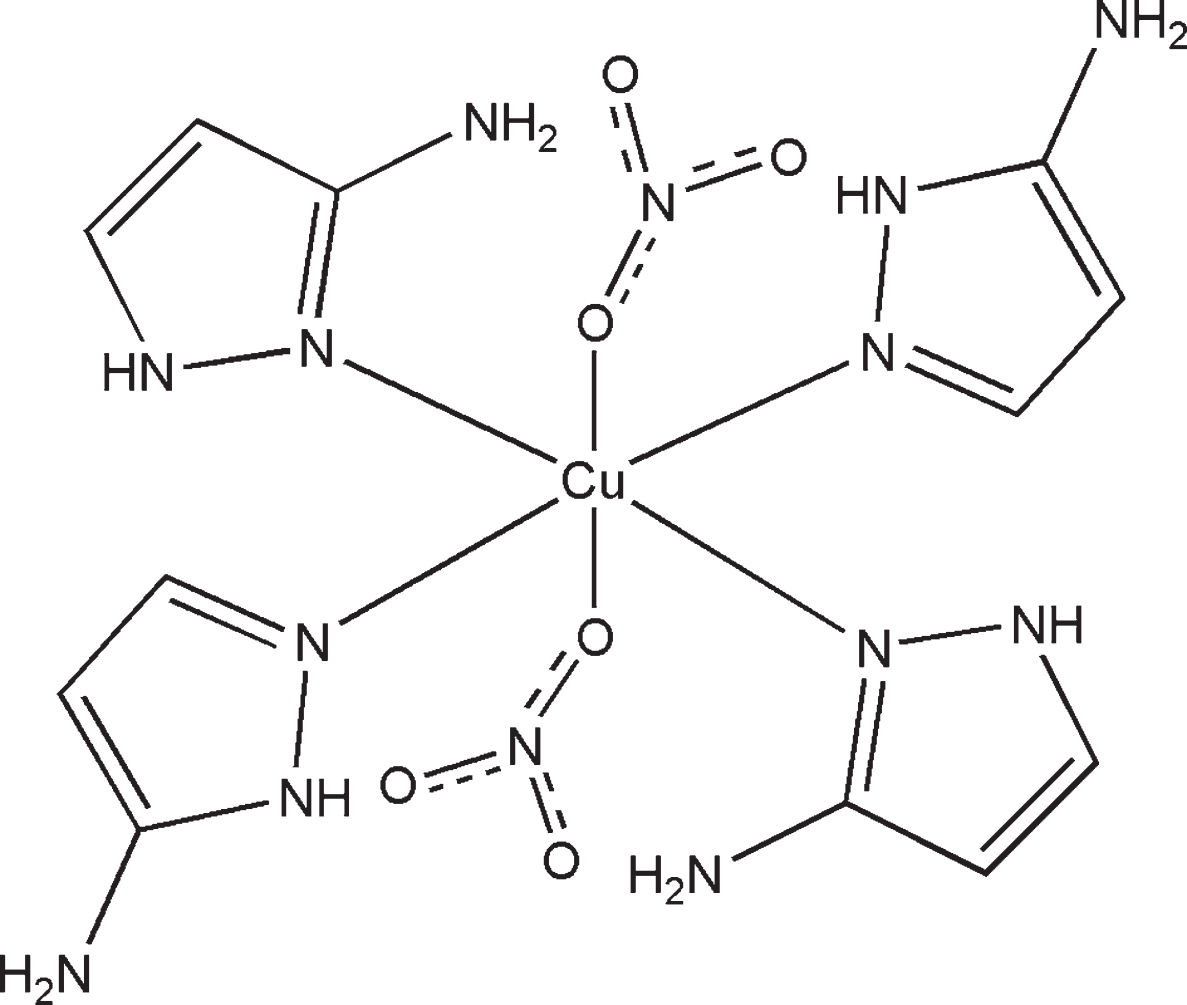


## 2. Structural commentary

**Figure 1:**
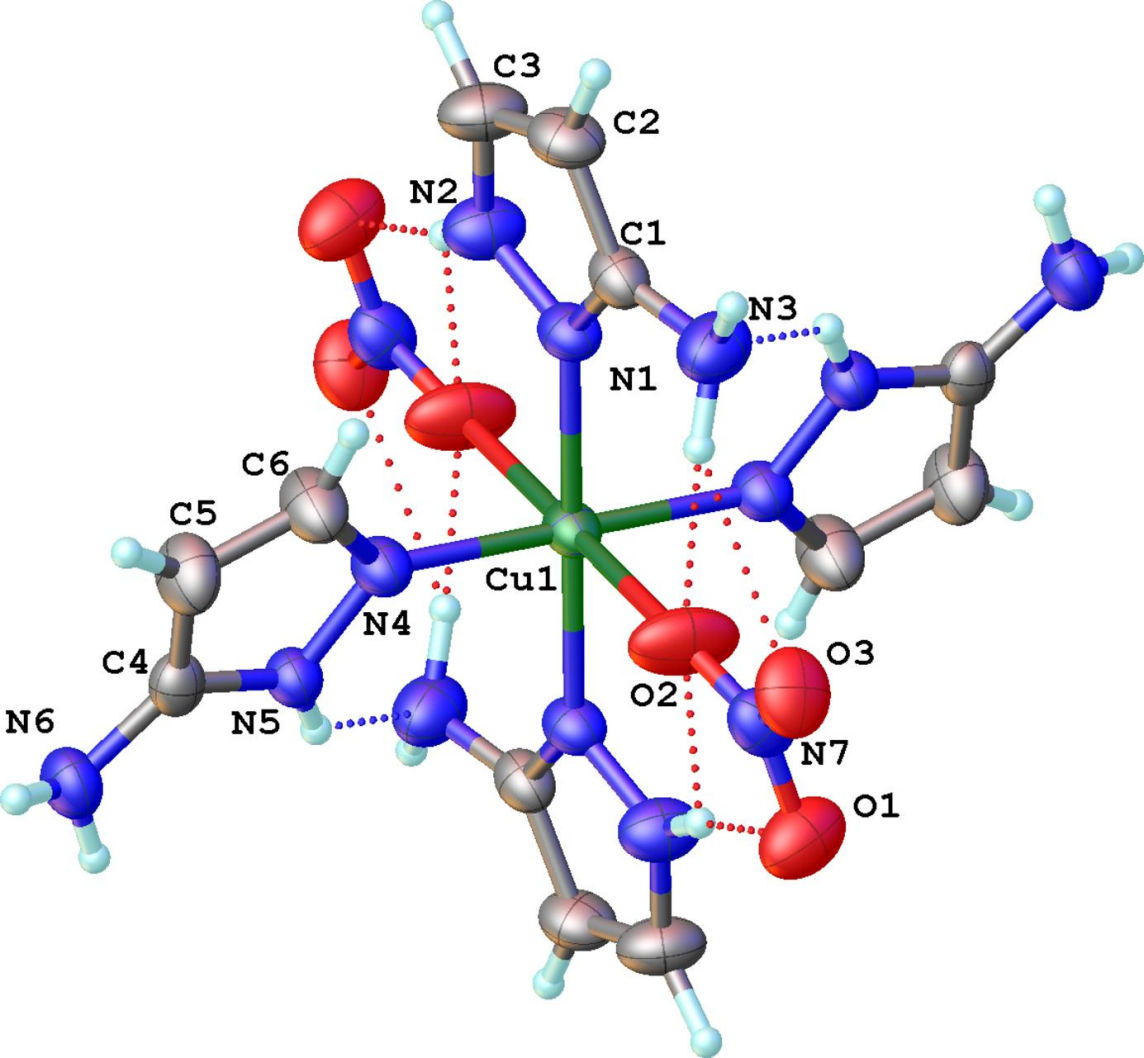
The mol­ecular structure of the title compound showing the atom-labeling scheme and displacement ellipsoids drawn at the 50% probability level. Intra­molecular N—H⋯O hydrogen bonds are shown as red dotted lines and N—H⋯N hydrogen bonds as blue dotted lines. Non-labeled atoms are generated by inversion symmetry [symmetry code: (i) 1 − *x*, −*y*, 1 − *z*].

The title compound is a mol­ecular coordination compound, where the central Cu^II^ atom is situated at an inversion center (Wyckoff position 2*c* of space group *P*2_1_/*n*) with an octa­hedral [N_4_O_2_] coordination environment. The axial positions are occupied by two oxygen atoms from nitrato ligands [Cu1—O2 = 2.5544 (19) Å, which is a typical value observed in Cu—(nitrato)^−^ complexes] and the equatorial positions are occupied by four pyridine-like nitro­gen atoms of 3-amino­pyrazole (Fig. 1 ▸). Two of the four 3-amino­pyrazole ligands coordinate with the N^1^ atom [Cu1—N4 = 1.975 (2) Å] while the other two coordinate with the N^2^ atom [Cu1—N1 = 2.0331 (17) Å]. The different type of N-coordination in the title compound is an illustrative example of tautomerism in 3-amino­pyrazole. This effect leads to the formation of more complex and diverse frameworks and expands the potential number of possible coordination compounds that can be formed in comparison with only one type of ligand. The notable difference in the Cu—O and Cu—N bond lengths leads to an elongation of the coordination octa­hedron, which is associated with the Jahn–Teller effect that is commonly observed for Cu^II^ complexes. The length distortion parameter *ζ* = |(Cu – *L*_i_) – <Cu – *L*>| (where *L* = ligand) for this structure is 1.468 Å. The deviation from an ideal octa­hedron for twelve *cis*-*L*—Cu—*L* angles can be described by the octa­hedral distortion parameter *Σ* = |90° – α_i_| = 17.33°. Pyrazole rings with the same type of coordination are located in one plane, while the angle between pyrazole rings with different types of coordination is 98.67 (11)°. The angle between the CuN_4_ and (N4/N5/C4–C6) planes is 16.9 (1)° while between the CuN_4_ and (N1/N2/C1–C3) planes, the corresponding angle is 101.48 (10)°. Intra­molecular hydrogen bonds stabilize the mol­ecular structure and include N—H⋯O contacts between 3-amino­pyrazole mol­ecules and the O atoms of the nitrato ligand as well as N—H⋯N contacts between the pyrrole-like N atom of one of the organic ligands and the amino group of another 3-amino­pyrazole ligand (Fig. 1 ▸, Table 1 ▸).

## 3. Supra­molecular features

**Figure 2:**
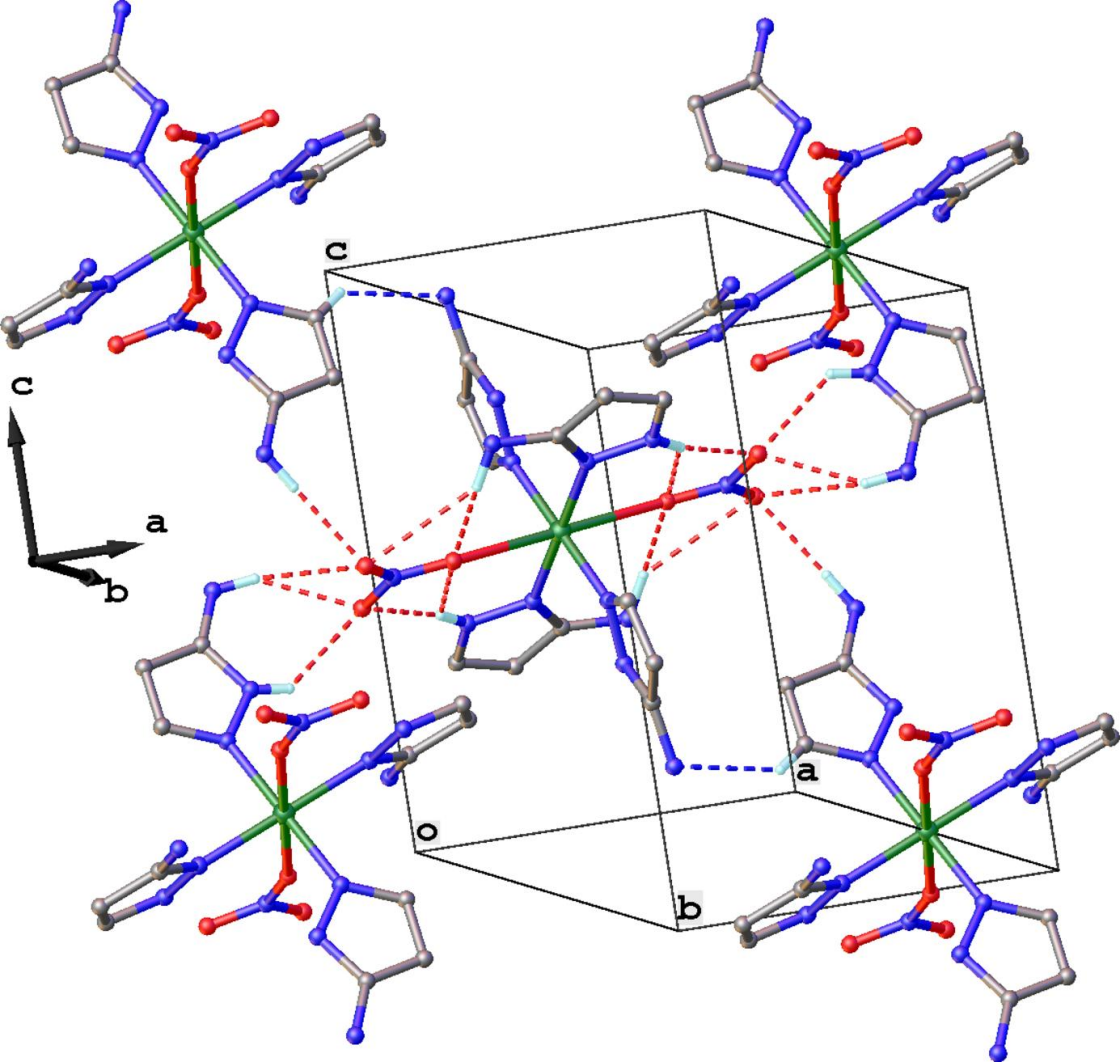
Supra­molecular packing of the title compound showing the extended network of weak inter­actions: N—H⋯O contacts are shown as red dashed lines and C—H⋯N contacts as blue dashed lines. N—H⋯N contacts and H atoms not involved in hydrogen bonding are omitted for clarity.

Mol­ecules of the title coordination compound inter­act with each other through a set of inter­molecular inter­actions, creating a supra­molecular tri-periodic network (Fig. 2 ▸). Inter­molecular hydrogen bonds include N—H⋯O contacts between 3-amino­pyrazole ligands and nitrate anions of a neighboring complex, as well as weak C—H⋯N contacts (Fig. 2 ▸). Numerical data of these hydrogen-bonding inter­actions is collated in Table 1 ▸.

## 4. Hirshfeld surface analysis

**Figure 3:**
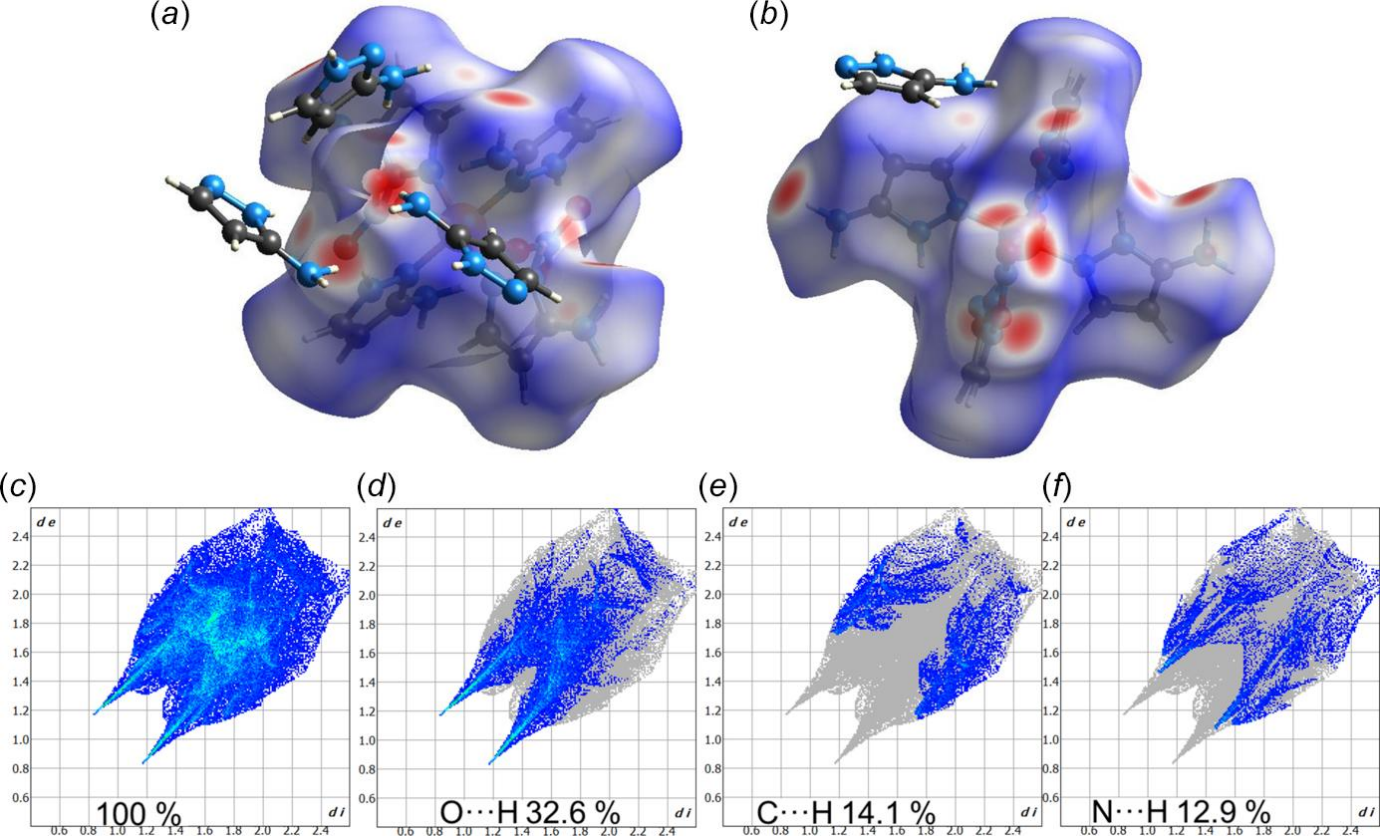
(*a*), (*b*) Hirshfeld surface of the title compound plotted over *d*_norm_. Neighboring mol­ecules creating contacts with the surface are shown as ‘balls and sticks’; the regions with the strongest inter­molecular inter­actions are plotted in red. (*c*) Hirshfeld surface fingerprint plots of the title compound showing the contribution of all inter­actions (100%) and those delineated into (*d*) O⋯H, (*e*) N⋯H, and (*f*) C⋯H inter­actions.

A Hirshfeld surface analysis was performed using *CrystalExplorer* (Spackman *et al.*, 2021 ▸) with a standard resolution of the three-dimensional *d*_norm_ surfaces plotted over a fixed color scale. The associated two-dimensional fingerprint plots were also generated. The Hirshfeld surface of the title compound demonstrates the presence of strong inter­molecular N—H⋯O hydrogen bonds between coordinating nitrate anions and neighboring 3-amino­pyrazole mol­ecules, as shown in Fig. 3 ▸ *a* in red. Fig. 3 ▸ *b* additionally demonstrates the presence of much weaker C—H⋯N contacts. Fingerprint plots are given for contacts with the highest contribution to the structure (Fig. 3 ▸ *c*–*f*). The most important contributions for the crystal packing are from O⋯H (32.6%), C⋯H (14.1%) and N⋯H (12.9%) contacts. H⋯H inter­actions are not shown. The *d*_e_ and *d*_i_ values presented on the axes of the fingerprint plots are the distances to the closest external and inter­nal atoms from a selected point to the Hirshfeld surface. It is worth noting that the fingerprint plots highlight the most frequently occurring weak inter­actions within the structure, whereas the graphical depiction of the surface emphasizes the strongest inter­actions.

## 5. Database survey

According to a search of the Cambridge Structural Database (CSD, version 5.43, last update March 2022; Groom *et al.*, 2016 ▸), there are only two records of copper(II) complexes containing 3-amino­pyrazole as a ligand: TIXDAH (Świtlicka-Olszewska *et al.*, 2014 ▸) and QIJSAF (Wang *et al.*, 2014 ▸). TIXDAH is [Cu(C_2_O_4_)(3-amino­pyrazole)_2_]·3H_2_O, in which Cu^II^ has a square-pyramidal [N_3_O_2_] coordination environment. The basal positions are occupied by the O atoms of a bidentate oxalate anion and two ring N atoms of two amino­pyrazole ligands, and the apical positions by the N atom of the amino group of another amino­pyrazole ligand. Similar to the title compound, the amino­pyrazole mol­ecules display different types of coordination – with N^1^ or N^2^ atoms. QIJSAF is [Cu(3-amino­pyrazole)(2,6-pyridinedi­carboxyl­ato)(H_2_O)]·H_2_O, in which Cu^II^ has a distorted octa­hedral [N_2_O_4_] environment. The equatorial positions are occupied by one N^2^-coordinating 3-amino­pyrazole and a tridentate 2,6-pyridinedi­carboxyl­ate ligand while the axial positions are taken up by one water mol­ecule and one carboxyl­ate O atom of another ligand.

## 6. Synthesis and crystallization

20 mg (0.1 mmol) of Cu(NO_3_)_2_ in 200 µl of water were mixed with 42 mg (0.5 mmol) of 3-amino­pyrazole in 200 µl of water. The obtained solution was left to evaporate in air. Within 24 h, blue crystals were collected from the reaction mixture and kept in the mother solution prior to the X-ray measurement.

## 7. Refinement

Crystal data, data collection and structure refinement details are summarized in Table 2 ▸. All H atoms were found from a difference-Fourier map and refined isotropically with *U*_iso_(H) = 1.2*U*_eq_(C) or *U*_iso_(H) = 1.2*U*_eq_(N).

## Supplementary Material

Crystal structure: contains datablock(s) I. DOI: 10.1107/S2056989023009295/wm5699sup1.cif

Structure factors: contains datablock(s) I. DOI: 10.1107/S2056989023009295/wm5699Isup2.hkl

CCDC reference: 2302897

Additional supporting information:  crystallographic information; 3D view; checkCIF report

## Figures and Tables

**Table 1:** Hydrogen-bond geometry (Å, °)

*D*—H⋯*A*	*D*—H	H⋯*A*	*D*⋯*A*	*D*—H⋯*A*
N3—H3*A*⋯O3	0.96 (4)	2.71 (3)	3.495 (3)	139 (3)
N3—H3*A*⋯O2	0.96 (4)	1.92 (4)	2.849 (3)	162 (3)
N3—H3*B*⋯O3^i^	0.96 (4)	2.20 (4)	3.085 (3)	153 (3)
N6—H6*A*⋯O3^ii^	0.87 (4)	2.83 (3)	3.480 (3)	132 (3)
N6—H6*A*⋯O1^ii^	0.87 (4)	2.22 (4)	2.999 (3)	149 (3)
N6—H6*B*⋯O3^iii^	0.88 (4)	2.14 (4)	3.019 (3)	176 (3)
N2—H2⋯N7^iv^	0.82 (4)	2.60 (4)	3.378 (3)	159 (3)
N2—H2⋯O1^iv^	0.82 (4)	2.10 (4)	2.908 (3)	171 (4)
N2—H2⋯O2^iv^	0.82 (4)	2.40 (4)	2.999 (3)	131 (3)
N5—H5⋯O1^ii^	0.80 (4)	2.47 (4)	3.093 (3)	136 (3)
N5—H5⋯N3^iv^	0.80 (4)	2.82 (4)	3.462 (3)	139 (3)
C6—H6⋯N6^v^	0.87 (4)	2.70 (4)	3.438 (3)	143 (3)

Symmetry codes: (i) 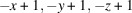 ; (ii) 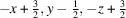 ; (iii) 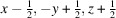 ; (iv) 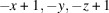 ; (v) 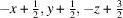 .

**Table 2:** Experimental details

Crystal data	
Chemical formula	[Cu(NO_3_)_2_(C_3_H_5_N_3_)_4_]
*M*_r_	519.96
Crystal system, space group	Monoclinic, *P*2_1_/*n*
Temperature (K)	200
*a*, *b*, *c* (Å)	8.83222 (18), 9.9714 (2), 12.1043 (2)
β (°)	97.6408 (19)
*V* (Å^3^)	1056.55 (4)
*Z*	2
Radiation type	Cu *K*α
μ (mm^−1^)	2.05
Crystal size (mm)	0.10 × 0.10 × 0.05

Data collection	
Diffractometer	XtaLAB Synergy, Dualflex, HyPix
Absorption correction	Multi-scan (*CrysAlis PRO*; Rigaku OD, 2023 ▸)
*T*_min_, *T*_max_	0.631, 1.000
No. of measured, independent and observed [*I* > 2σ(*I*)] reflections	6493, 2023, 1903
*R*_int_	0.025
(sin θ/λ)_max_ (Å^−1^)	0.629

Refinement	
*R*[*F* ^2^ > 2σ(*F* ^2^)], *wR*(*F* ^2^), *S*	0.038, 0.114, 1.11
No. of reflections	2023
No. of parameters	181
H-atom treatment	Only H-atom coordinates refined
Δρ_max_, Δρ_min_ (e Å^−3^)	0.45, −0.43

Computer programs: *CrysAlis PRO* (Rigaku OD, 2023 ▸), *SHELXT* (Sheldrick, 2015*a* ▸), *SHELXL* (Sheldrick, 2015*b* ▸), and *OLEX2* (Dolomanov *et al.*, 2009 ▸).

## supplementary crystallographic information

### Crystal data

**Table:**

[Cu(NO_3_)_2_(C_3_H_5_N_3_)_4_]	*F*(000) = 534
*M**_r_* = 519.96	*D*_x_ = 1.634 Mg m^−^^3^
Monoclinic, *P*2_1_/*n*	Cu *K*α radiation, λ = 1.54184 Å
*a* = 8.83222 (18) Å	Cell parameters from 4669 reflections
*b* = 9.9714 (2) Å	θ = 5.0–75.6°
*c* = 12.1043 (2) Å	µ = 2.05 mm^−^^1^
β = 97.6408 (19)°	*T* = 200 K
*V* = 1056.55 (4) Å^3^	Prism, clear light blue
*Z* = 2	0.10 × 0.10 × 0.05 mm

### Data collection

**Table:**

XtaLAB Synergy, Dualflex, HyPix diffractometer	2023 independent reflections
Radiation source: micro-focus sealed X-ray tube, PhotonJet (Cu) X-ray Source	1903 reflections with *I* > 2σ(*I*)
Mirror monochromator	*R*_int_ = 0.025
Detector resolution: 10.0000 pixels mm^-1^	θ_max_ = 76.0°, θ_min_ = 5.8°
ω scans	*h* = −10→10
Absorption correction: multi-scan (CrysAlisPro; Rigaku OD, 2023)	*k* = −12→11
*T*_min_ = 0.631, *T*_max_ = 1.000	*l* = −14→14
6493 measured reflections	

### Refinement

**Table:**

Refinement on *F*^2^	0 restraints
Least-squares matrix: full	Hydrogen site location: difference Fourier map
*R*[*F*^2^ > 2σ(*F*^2^)] = 0.038	Only H-atom coordinates refined
*wR*(*F*^2^) = 0.114	*w* = 1/[σ^2^(*F*_o_^2^) + (0.0689*P*)^2^ + 0.4789*P*] where *P* = (*F*_o_^2^ + 2*F*_c_^2^)/3
*S* = 1.11	(Δ/σ)_max_ < 0.001
2023 reflections	Δρ_max_ = 0.45 e Å^−^^3^
181 parameters	Δρ_min_ = −0.43 e Å^−^^3^

### Special details

**Table:**

Geometry. All esds (except the esd in the dihedral angle between two l.s. planes) are estimated using the full covariance matrix. The cell esds are taken into account individually in the estimation of esds in distances, angles and torsion angles; correlations between esds in cell parameters are only used when they are defined by crystal symmetry. An approximate (isotropic) treatment of cell esds is used for estimating esds involving l.s. planes.

### Fractional atomic coordinates and isotropic or equivalent isotropic displacement parameters (Å^2^)

**Table:**

	*x*	*y*	*z*	*U*_iso_*/*U*_eq_	
Cu1	0.500000	0.000000	0.500000	0.03217 (18)	
N5	0.4258 (2)	−0.05809 (18)	0.72784 (15)	0.0330 (4)	
N4	0.3930 (2)	0.01620 (18)	0.63262 (17)	0.0353 (4)	
O3	0.7519 (2)	0.40655 (17)	0.58858 (17)	0.0561 (5)	
N7	0.7646 (2)	0.28338 (19)	0.59003 (16)	0.0417 (5)	
O1	0.8905 (2)	0.2305 (2)	0.6228 (2)	0.0625 (5)	
C1	0.3111 (3)	0.2405 (2)	0.38736 (18)	0.0359 (5)	
N1	0.3312 (2)	0.11018 (18)	0.41243 (15)	0.0354 (4)	
O2	0.6506 (3)	0.2110 (2)	0.5642 (2)	0.0702 (7)	
N6	0.3538 (3)	−0.0813 (2)	0.90865 (17)	0.0442 (5)	
N2	0.1924 (2)	0.0519 (2)	0.38069 (19)	0.0469 (5)	
C4	0.3441 (3)	−0.0178 (2)	0.80771 (19)	0.0341 (5)	
C5	0.2547 (3)	0.0873 (3)	0.7639 (2)	0.0488 (6)	
C6	0.2881 (3)	0.1034 (3)	0.6570 (2)	0.0498 (6)	
C2	0.1602 (3)	0.2639 (3)	0.3389 (2)	0.0443 (5)	
C3	0.0907 (3)	0.1423 (3)	0.3363 (2)	0.0516 (6)	
N3	0.4272 (3)	0.3290 (2)	0.4016 (2)	0.0530 (5)	
H3A	0.499 (4)	0.306 (4)	0.465 (3)	0.064*	
H3B	0.400 (4)	0.422 (4)	0.393 (3)	0.064*	
H6A	0.434 (4)	−0.132 (4)	0.927 (3)	0.064*	
H6B	0.323 (4)	−0.034 (4)	0.962 (3)	0.064*	
H2	0.177 (4)	−0.029 (4)	0.385 (3)	0.064*	
H5	0.488 (4)	−0.116 (4)	0.731 (3)	0.064*	
H3	−0.016 (4)	0.117 (3)	0.296 (3)	0.064*	
H6	0.254 (4)	0.165 (4)	0.609 (3)	0.064*	
H2A	0.123 (4)	0.346 (4)	0.307 (3)	0.064*	
H5A	0.190 (4)	0.135 (4)	0.798 (3)	0.064*	

### Atomic displacement parameters (Å^2^)

**Table:**

	*U*^11^	*U*^22^	*U*^33^	*U*^12^	*U*^13^	*U*^23^
Cu1	0.0362 (3)	0.0295 (3)	0.0316 (3)	0.01035 (15)	0.00732 (19)	0.00466 (15)
N5	0.0352 (9)	0.0324 (9)	0.0321 (9)	0.0042 (7)	0.0069 (7)	0.0022 (7)
N4	0.0377 (9)	0.0340 (9)	0.0347 (9)	0.0083 (7)	0.0068 (8)	0.0038 (7)
O3	0.0711 (12)	0.0293 (8)	0.0706 (12)	−0.0027 (8)	0.0197 (10)	0.0045 (8)
N7	0.0518 (12)	0.0323 (10)	0.0403 (10)	−0.0068 (8)	0.0041 (8)	0.0013 (7)
O1	0.0491 (10)	0.0432 (10)	0.0939 (15)	−0.0020 (8)	0.0044 (10)	0.0070 (10)
C1	0.0423 (11)	0.0329 (10)	0.0338 (10)	0.0117 (9)	0.0103 (8)	0.0042 (8)
N1	0.0383 (9)	0.0335 (9)	0.0344 (9)	0.0104 (7)	0.0049 (7)	0.0037 (7)
O2	0.0666 (13)	0.0423 (10)	0.0904 (16)	−0.0113 (9)	−0.0318 (11)	−0.0032 (10)
N6	0.0498 (11)	0.0483 (11)	0.0363 (10)	0.0020 (9)	0.0126 (8)	0.0018 (9)
N2	0.0469 (11)	0.0328 (10)	0.0576 (12)	0.0049 (8)	−0.0053 (9)	0.0004 (9)
C4	0.0344 (11)	0.0327 (10)	0.0362 (11)	−0.0059 (8)	0.0080 (9)	−0.0030 (8)
C5	0.0554 (14)	0.0444 (13)	0.0514 (14)	0.0156 (11)	0.0244 (11)	0.0008 (11)
C6	0.0563 (15)	0.0474 (13)	0.0488 (13)	0.0231 (12)	0.0190 (11)	0.0106 (11)
C2	0.0482 (13)	0.0395 (12)	0.0437 (12)	0.0166 (10)	−0.0001 (10)	0.0048 (10)
C3	0.0455 (14)	0.0462 (14)	0.0579 (15)	0.0099 (11)	−0.0121 (12)	−0.0018 (11)
N3	0.0438 (12)	0.0467 (12)	0.0680 (15)	0.0033 (9)	0.0055 (10)	0.0122 (10)

### Geometric parameters (Å, º)

**Table:**

Cu1—N4	1.9748 (19)	N6—C4	1.369 (3)
Cu1—N4^i^	1.9748 (19)	N6—H6A	0.87 (4)
Cu1—N1	2.0331 (17)	N6—H6B	0.88 (4)
Cu1—N1^i^	2.0332 (17)	N2—C3	1.334 (3)
N5—N4	1.368 (3)	N2—H2	0.82 (4)
N5—C4	1.343 (3)	C4—C5	1.375 (3)
N5—H5	0.80 (4)	C5—C6	1.375 (4)
N4—C6	1.332 (3)	C5—H5A	0.88 (3)
O3—N7	1.233 (3)	C6—H6	0.87 (4)
N7—O1	1.247 (3)	C2—C3	1.358 (4)
N7—O2	1.245 (3)	C2—H2A	0.94 (4)
C1—N1	1.340 (3)	C3—H3	1.03 (3)
C1—C2	1.403 (3)	N3—H3A	0.96 (4)
C1—N3	1.347 (3)	N3—H3B	0.96 (4)
N1—N2	1.364 (3)		
			
N4—Cu1—N4^i^	180.0	H6A—N6—H6B	116 (3)
N4—Cu1—N1^i^	91.01 (7)	N1—N2—H2	123 (3)
N4—Cu1—N1	88.99 (7)	C3—N2—N1	111.0 (2)
N4^i^—Cu1—N1	91.00 (7)	C3—N2—H2	126 (3)
N4^i^—Cu1—N1^i^	89.00 (7)	N5—C4—N6	121.9 (2)
N1—Cu1—N1^i^	180.0	N5—C4—C5	106.7 (2)
N4—N5—H5	120 (2)	N6—C4—C5	131.4 (2)
C4—N5—N4	111.75 (18)	C4—C5—H5A	127 (2)
C4—N5—H5	128 (2)	C6—C5—C4	105.5 (2)
N5—N4—Cu1	124.74 (14)	C6—C5—H5A	127 (2)
C6—N4—Cu1	130.97 (17)	N4—C6—C5	112.0 (2)
C6—N4—N5	104.04 (19)	N4—C6—H6	120 (2)
O3—N7—O1	120.1 (2)	C5—C6—H6	128 (2)
O3—N7—O2	120.3 (2)	C1—C2—H2A	125 (2)
O2—N7—O1	119.5 (2)	C3—C2—C1	105.2 (2)
N1—C1—C2	110.2 (2)	C3—C2—H2A	129 (2)
N1—C1—N3	122.1 (2)	N2—C3—C2	108.4 (2)
N3—C1—C2	127.5 (2)	N2—C3—H3	123 (2)
C1—N1—Cu1	135.03 (16)	C2—C3—H3	127.7 (19)
C1—N1—N2	105.19 (18)	C1—N3—H3A	111 (2)
N2—N1—Cu1	119.06 (14)	C1—N3—H3B	116 (2)
C4—N6—H6A	117 (2)	H3A—N3—H3B	117 (3)
C4—N6—H6B	115 (2)		
			
Cu1—N4—C6—C5	−173.70 (19)	N6—C4—C5—C6	−176.8 (3)
Cu1—N1—N2—C3	172.49 (18)	C4—N5—N4—Cu1	174.56 (15)
N5—N4—C6—C5	0.5 (3)	C4—N5—N4—C6	−0.1 (3)
N5—C4—C5—C6	0.6 (3)	C4—C5—C6—N4	−0.7 (3)
N4—N5—C4—N6	177.4 (2)	C2—C1—N1—Cu1	−170.29 (17)
N4—N5—C4—C5	−0.3 (3)	C2—C1—N1—N2	−0.6 (2)
C1—N1—N2—C3	0.8 (3)	N3—C1—N1—Cu1	14.0 (3)
C1—C2—C3—N2	0.3 (3)	N3—C1—N1—N2	−176.3 (2)
N1—C1—C2—C3	0.2 (3)	N3—C1—C2—C3	175.6 (3)
N1—N2—C3—C2	−0.7 (3)		

Symmetry code: (i) −*x*+1, −*y*, −*z*+1.

### Hydrogen-bond geometry (Å, º)

**Table:**

*D*—H···*A*	*D*—H	H···*A*	*D*···*A*	*D*—H···*A*
N3—H3*A*···O3	0.96 (4)	2.71 (3)	3.495 (3)	139 (3)
N3—H3*A*···O2	0.96 (4)	1.92 (4)	2.849 (3)	162 (3)
N3—H3*B*···O3^ii^	0.96 (4)	2.20 (4)	3.085 (3)	153 (3)
N6—H6*A*···O3^iii^	0.87 (4)	2.83 (3)	3.480 (3)	132 (3)
N6—H6*A*···O1^iii^	0.87 (4)	2.22 (4)	2.999 (3)	149 (3)
N6—H6*B*···O3^iv^	0.88 (4)	2.14 (4)	3.019 (3)	176 (3)
N2—H2···N7^i^	0.82 (4)	2.60 (4)	3.378 (3)	159 (3)
N2—H2···O1^i^	0.82 (4)	2.10 (4)	2.908 (3)	171 (4)
N2—H2···O2^i^	0.82 (4)	2.40 (4)	2.999 (3)	131 (3)
N5—H5···O1^iii^	0.80 (4)	2.47 (4)	3.093 (3)	136 (3)
N5—H5···N3^i^	0.80 (4)	2.82 (4)	3.462 (3)	139 (3)
C6—H6···N6^v^	0.87 (4)	2.70 (4)	3.438 (3)	143 (3)

Symmetry codes: (i) −*x*+1, −*y*, −*z*+1; (ii) −*x*+1, −*y*+1, −*z*+1; (iii) −*x*+3/2, *y*−1/2, −*z*+3/2; (iv) *x*−1/2, −*y*+1/2, *z*+1/2; (v) −*x*+1/2, *y*+1/2, −*z*+3/2.
